# 

**Published:** 1917-08-04

**Authors:** 


### CONTENTS.

| | PAGE |
|---|---|
| **Medical Politics...** | 349 |
| **Rotten Food for Londoners, Why?** | 350 |
| **Hospital and Institutional News ...** | 351 |
| Undeserved and Ill-founded Criticism—Plastic Surgery at Sidcup—The Doom of the Quack—The Military Out-patient Problem—The Unfit and the Army—The Affairs of Auxiliary Hospitals—Doctors and Taxi-cabs—The Prince of Wales' Hospital for Limbless Sailors and Soldiers, Cardiff—A Medical Revolt—Lightening the Medical Duties at Bermondsey—Australian Doctors and Compulsion—The Soldier's Mother—Manchester Lock Hospital's New Scheme—Hospitals on Ships—Retford Hospital to be Rebuilt—The Pilkington Special Hospital—The Cyril Clayden Bed—Mortality in War Hospitals—Soldiers and Fancy-work—The Management of Sanatoria—Tuberculous Milk in London—Ferrary's Hospital Stamps—Brighton and Child Welfare—Have the Blind a Sixth Sense?—Admission to Isolation Hospitals—Healthy London—The *Searchlight* and *Southern Cross*—This Week's Drug Market—To Our Readers. | |
| **Recruiting by the New Method ...** | 357 |
| Civilian Doctors to Replace Military Officers. | |
| **The Institutional Library ...** | 359 |
| **The Value of Voluntary Effort ...** | 361 |
| The Leicester Home for Neurasthenic Soldiers. | |
| **Parliament and the Profession ...** | 361 |
| **The Editor's Letter-Box ...** | 362 |
| Nursing Affairs in Ireland. | |
| **The Matrons' and Sisters' Department** | 363 |
| Matrons in Council: Sparks from a Nurse's Anvil. | |
| Round the Hospitals. | |
| The King with Australian Nurses—Nurses' Bravery on the Field—Miss Amy Hughes's Diffidence—Nursing Affairs in Ireland—That "Choker" Collar Again. | |
| **The College of Nursing, Limited** | 366 |
| Miss Matheson Speaks at Belfast. | |
| **The Book Market ...** | vi |
| **Medical Appointments ...** | vi |
| **Matrons' and Sisters' Appointments ...** | vi |
| **Gifts and Bequests ...** | vi |
| **The Institutional Worker ...** (Suppl.) | 1 |
| Diet, Occupations, and Nursing in Inebriates' Home: Answer to June Question. | |
| Question for August. | |
| Enquiries and Answers. | |
| War Matters. | |
| Employment and Training. | |
| The Housekeepers' Department. | |
| Miscellaneous. | |

